# Late Complication of Laparoscopic Sleeve Gastrectomy

**DOI:** 10.1155/2013/136153

**Published:** 2013-04-11

**Authors:** Anthony Dakwar, Ahmad Assalia, Iyad Khamaysi, Yoram Kluger, Ahmad Mahajna

**Affiliations:** ^1^Department of General Surgery, Rambam Health Care Campus, The Technion-Institute of Technology, P.O. Box 9602, Haifa 31096, Israel; ^2^Department of Gastroenterology and Hepatology, Rambam Health Care Campus, The Technion-Institute of Technology, P.O. Box 9602, Haifa 31096, Israel

## Abstract

Laparoscopic sleeve gastrectomy (LSG) is gaining popularity for the treatment of morbid obesity. It is a simple, low-cost procedure resulting in significant weight loss within a short period of time. LSG is a safe procedure with a low complication rate. The complications encountered nevertheless can result in morbidity and even mortality. The most significant complications are staple-line bleeding, stricture, and staple-line leak. The purpose of this paper is to present a patient who suffered from a staple-line leak presenting 16 months after LSG. Review of the current literature regarding this complication as well as outline of a strategy for the management of post-LSG gastric leaks is suggested.

## 1. Introduction

Morbid obesity has become a common epidemic in the western cultures and is slowly spreading to the rest of the world. By year 2025, it is estimated that 40% of American society will be morbidly obese [1]. Although many dietary therapies are available, patients seem to be most responsive to surgical intervention. 

Current surgical strategies consist of laparoscopic adjustable gastric banding (LAGB), laparoscopic sleeve gastrectomy (LSG), laparoscopic Roux-en-Y gastric bypass (LRYGBP), and laparoscopic biliopancreatic diversion with duodenal switch (LBPD-DS) [1]. 

LSG has become popular due to its simplicity and low complication rate. LSG was first performed in 2000, by Gagner and Patterson, as part of a duodenal switch procedure [2]. Regan et al. suggested sleeve gastrectomy as the first step in gastric bypass surgery as an alternative procedure in high-risk obese patients to decrease mortality and morbidity [3]. Currently, many surgeons are considering LSG as a stand-alone procedure that offers a substantial weight loss for the obese patient [4, 5]. It has been shown to be as effective as reducing excess weight by 60–70% within 3 years [5]. 

The physiological and anatomical reasoning supporting the efficacy of LSG is attributed to the reduction of total gastric capacity, illustrating a restrictive effect [4, 6–8]. In addition, an orexigenic/anorexigenic hormonal modification is evident due to the removal of fundal ghrelin-producing cells [4, 6].

LSG is a simple surgical procedure resulting in low complication rate with insignificant long-term nutritional deficiencies, especially when compared to the other alternative, more aggressive bariatric procedures. Its complications consist mainly of staple-line bleeding, strictures (usually located at the middle or distal portion of the residual stomach), and the most severe, dangerous complication being staple-line leaks [9]. The reported gastric leak rates from the sleeve staple line are 1.4–2.5% for primary sleeve gastrectomies and 16–20% for reoperative surgery where a previous gastric operation has been performed [10–13].

The aim of this paper is to present a unique presentation of late gastric leak and to provide a review of current approach to management and treatment of gastric leaks after LSG. 

## 2. Case Report

### 2.1. Surgical Technique

Previous publications have meticulously outlined in detail the procedure of LSG [14–16]. This procedure started with administration of 15 mmHg within peritoneum. 4 trocars are placed: one 15 mm, two 10 mm, and one 5 mm. A 32F bougie is introduced into stomach by anesthesiologist to help guide the surgeon in making an equivalent division. Beginning 2-3 cm proximal to the pylorus up until 1 cm distal of the angle of His, the stomach is divided using an Endo GIA stapler (Ethicon Endo-surgery, Cincinnati, OH, USA) leaving a gastric pouch of 60–80 mL capacity. Prior to stapling, vessels of greater curvature are divided using LigaSure device (Valleylab, Tyco Healthcare Group Lp, Boulder, CO 80301-3299, USA). 

### 2.2. Patient

A 42-year-old male, presented with long-standing morbid obesity as a BMI of 45 weighing 148 kilograms. His comorbidities included hypertension treated with enalapril. Prior surgical history was a LAGB in 2001 with a BMI of 40 and a weight loss of 35 kilograms. This surgery resulted in decreasing his weight from 140 to 105 kilograms within a time setting of 2 years. In March of 2009, due to regaining of weight, the adjustable gastric band was removed in preparation for LSG. The LSG, with reinforcement sutures, performed two months later was uneventful; the patient was hospitalized for 2 days with no signs or symptoms of postoperative complications. He was subsequently discharged home. The patient attended 3 postoperative visits within the year after the procedure; all followups were unremarkable. He lost 55 kilograms of excess weight. Note that during this period the patient did not undergo endoscopic examination. 

Sixteen months after LSG, the patient presented to the hospital with a fever of 39°C, left upper abdominal pain, and chills for the duration of two weeks. Laboratory findings were unremarkable except for leukocytosis of 21.7 × 10^9^/L. Physical examination revealed abdominal tenderness. 

**Figure 1:**
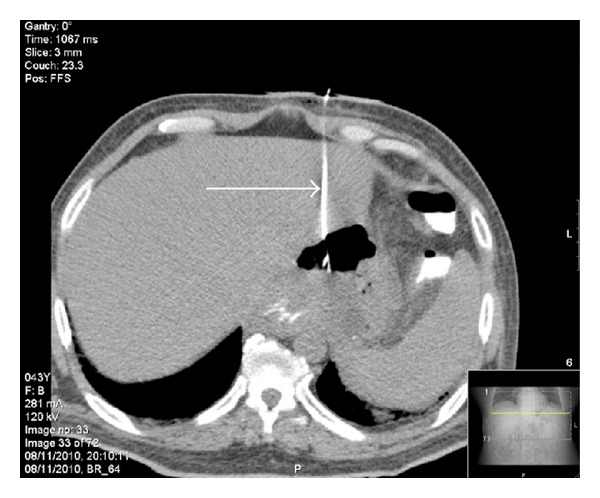
CT scan detected an abscess with dimensions of 4.9 × 9.0 cm located along the subdiaphragmatic border near the gastroesophageal junction. CT identified no gastric leak. The abscess was drained with a 7-French drainage tube (arrow).

CT scan revealed a 4.9 × 9.0 cm abscess with air-fluid level along the subdiaphragmatic border near the gastroesophageal junction (Figure 1). No gastric leak was noticed. The abscess was drained with a 7-French drainage tube. 200 mL of purulent material was drained.

**Figure 2:**
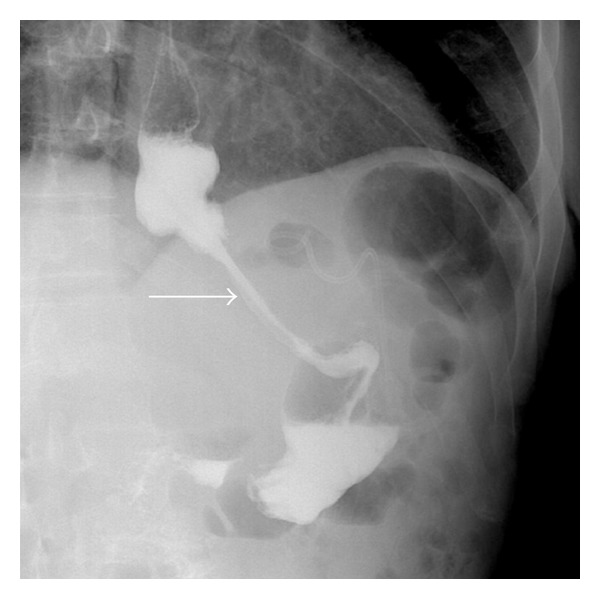
Gastrografin fluoroscopy on the upper gastrointestinal tract; no leak was identified (arrow pointing to “Sleeve”).

**Figure 3:**
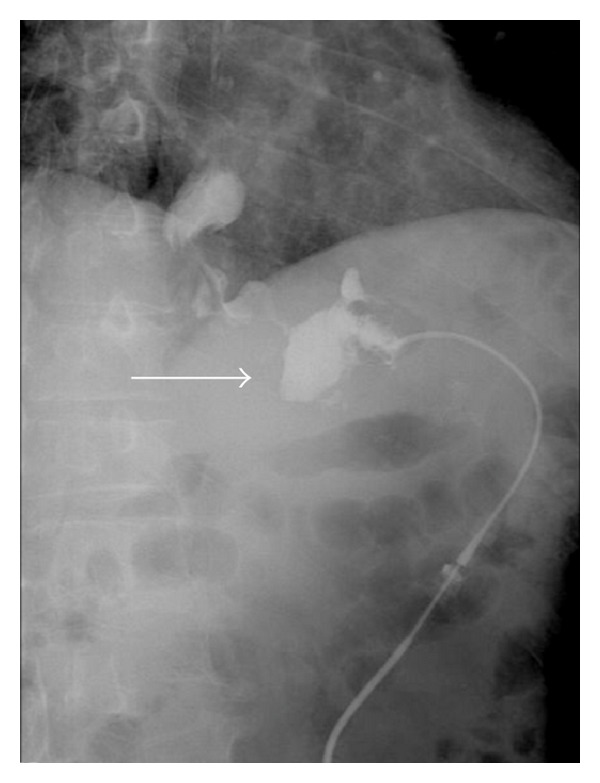
Gastrografin fluoroscopy performed through the drainage tube imitating a “gastrografin fistulography” (tubogram) image, successfully illustrating the gastric leak (arrow).

**Figure 4:**
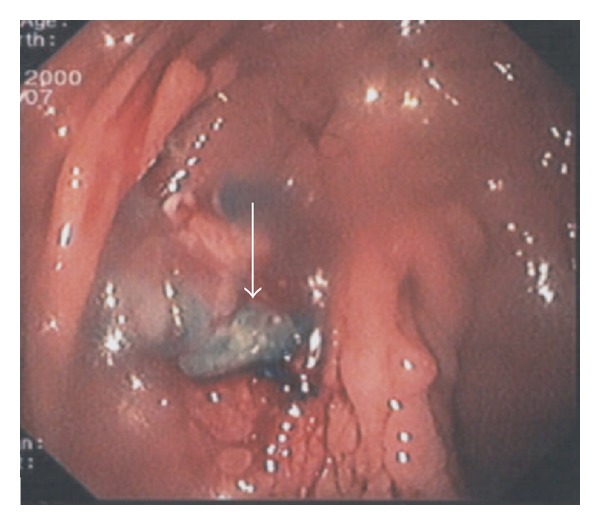
On upper endoscopy with MB test, the fistula orifice was clearly identified and located 2 cm distal to squamocolumnar junction (*Z*-line) (arrow).

**Figure 5:**
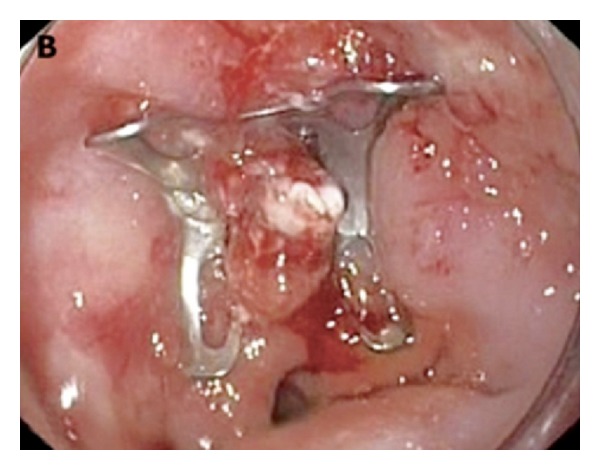
The fistula was hermitically sealed by deployment of a newly designed 10 mm over-the-scope metallic clip.

Gastrografin swallow fluoroscopy did not identify a leak (Figure 2). Gastrografin fluoroscopy was performed through the drainage tube imitating a “gastrografin fistulograph” (tubogram) image, and it successfully illustrated the gastric leak (Figure 3). On upper endoscopy with methylene blue test, the fistula orifice was clearly identified and located 2 cm distal to squamocolumnar junction (Z-line) (Figure 4). The fistula was hermitically sealed by deployment of a newly designed 10 mm over-the-scope metallic clip ( Ovesco's product, Ovesco Endoscopy GmbH, Tuebingen, Germany) (Figure 5).

Three weeks later, patient returned with a presentation of slight left flank pain. CT imaging revealed clips in place with no evidence of recurrence of leak. Drain was subsequently removed upon same visit.

## 3. Discussion

LSG is becoming a very popular stand-alone surgical procedure in providing treatment for morbid obesity. Of the few complications, most common and important are staple-line bleeding, strictures (usually located at the middle or distal portion of the residual stomach), and the most severe, dangerous complication being staple-line leaks [9, 16–18]. Reports of gastric leak after LSG have been within the range of 0.7% to 5.3% (mean 2.3%) [17–24]. Gastric leak is mostly likely to occur along the proximal third of the stomach, close to the gastroesophageal junction due to high intragastric pressure with impaired peristaltic activity and ischemia [16, 25].

Csendes et al. have developed a system of classification for gastric leaks based on three parameters: time of appearance after surgery, magnitude or severity, and location. The three categories are early leaks that appear 1–4 days after surgery, intermediate leaks that appear 5–9 days after surgery, and late leaks that appear at day 10 or later after surgery [9]. This case report is unique in the fact that it represents a rare long-term presentation of gastric leak after LSG. It shows that the followup for LSG complications should be prolonged, especially in patients with increased risk factors. The severity of gastric leaks is divided into type I: subclinical appearing as a local leak without spillage or dissemination and type II: leaks resulting in dissemination or diffusion into the abdominal or pleural cavity [9]. It has been noted that extraluminal gastric leaks, if not treated promptly and correctly, may lead to gastric-cutaneous fistula, peritonitis, abscess, sepsis, organ failure, and death [26].

The cause of a gastric leak is indicative of some abnormality or failure of normal healing process of tissue. There is a general agreement that local risk factors contributing to a leak are impaired suture line healing due to staple dehiscence, poor blood flow, and infection. These risk factors contribute to decrease in oxygen and subsequent ischemia to the tissue [9, 16, 25, 27]. Csendes et al. state direct doubt that staple line dehiscence is a likely risk factor due the efficiency of the ENDOGIA apparatus, which lays 3 lines of staples [9]. Some claim that the actual etiology of these leaks is due to some form of thermal damage upon tissue from the laparoscopic tools such as the endostaple or electrocautery devices. Baker suggests two main category of leaks: classic ischemic leak that tend to appear between 5-6 days after surgery and mechanical tissular that tend to appear within 2 days after surgery [28]. In current case presentation of a gastric leak 16 months after LSG the exact mechanism is obscure. Diagnosis of a gastric leak can be difficult, as the presentation can vary from asymptomatic to severe septic shock. Usual symptoms may be of the septic nature: fever, tachycardia, tachypnea, leukocytosis, abdominal pain, and peritonitis. Burgos et al. report that the initial sign of early leak was tachycardia in a series of 7 leaks in 214 patients (3.3%) [16]. In another series of 9 leaks in 210 patients, Hamilton et al. claim that tachycardia >120 beats per minute (bpm) may be the most diagnostic sign of a gastric leak [29]. Csendes et al. reported that fever was the most important and clinical indicator of gastric leaks [9]. In their series of 16 gastric leaks in 343 patients (4.66%), consistent recording of fever was apparent in all 3 categories of leak: early, intermediate, and late. More interestingly, fever was the most common sign as well as earliest to be recognized, even before the confirmation of the presence of a leak through radiological technique. In the presented case, fever was the first and most consistent symptom noted throughout, adding to the notion that initial apparent symptoms are particularly of importance when reaching diagnosis. 

There are currently no protocol shows that how to manage and treat a gastric leak. However, from the literature, there is a collective agreement among the authors that timing of diagnosis plays an important role in deciding the invasiveness and urgency of treatment. Early diagnosis (<3 days) has been shown to have a better prognosis when treated immediately surgically: either laparoscopic or open washout, drainage placement, and resuturing of leak if tissue is still in early stages of inflammation. Late diagnosis can be treated more conservatively: placement of drain, enteral nutrition, NPO, high-dose proton pump inhibitor, and broad-spectrum antibiotics [9, 16, 25, 27, 30]. Serial fluoroscopic testing is recommended weekly to ensure proper healing as well as to indicate if more invasive treatment is required.

According to the First International Consensus Summit for Sleeve Gastrectomy, treatment of leak included early oversewing, drainage (CAT or open), endoscopic clipping, and persisting fistula requiring fibrin glue, stents, Roux-loop, and even total gastrectomy [5]. Nguyen et al. have shown success in treatment of gastric leaks with endoscopic stenting. Given that the stent can only provide proper sealing in proximal and mid-aspect gastric sleeve leaks, it should be considered as an option in treatment [31]. In most recent study, Bege et al. have shown success and suggested an approach to endoscopic management of postbariatric fistula complications. It consists of three stages: lavage and drainage of the perianastomotic fluid (natural endoscopic transluminal endoscopic surgery “NOTES”), fistula diversion by placement of covered stent, and finally closure of fistula by clips or glue (either fibrin or cyanoacrylate) [32]. Bege et al. illustrated a safe and effective treatment modality towards complications of postbariatric procedures that encourages the initial treatment to be by endoscopic techniques and to avoid unnecessary surgery intervention. 

A main point that needs to be addressed is how can these leaks be avoided? Since the exact etiology of the majority of leaks cannot be defined confidently, surgery techniques should be considered as an area open to improvement. It is agreed upon that thermal damage induced by the laparoscopic devices may be a contributing factor to the development of gastric leaks. According to Baker and Armstrong, among many others, it is advisable to carefully compress the tissue being manipulated and to sustain the position in order to allow sufficient time for fluids to exit and for the staples to be placed with ease. A consensus of gentle compression for approximately 10 seconds should be enough time to reduce the trauma level to the tissue [28, 33]. 

## 4. Conclusion

In conclusion, LSG has been popular as a stand-alone treatment of choice for morbid obesity. It has been shown to be extremely successful in decreasing excess weight in patients within a short time. In addition, a short list of complications contributes to its attractiveness as a treatment. Among them, gastric leaks after LSG procedures can be a very serious, life-threatening complication that needs immediate attention. Currently, the literature has yet to define an absolute algorithm as to how to manage and treat gastric leaks; however, there is a consensus that timing of diagnosis, severity, and location all play a role in constructing a treatment plan. 

## References

[1] Kopelman PG (2000). Obesity as a medical problem. *Nature*.

[2] Gagner M, Patterson E (2000). Laparoscopic biliopancreatic diversion with duodenal switch. *Digestive Surgery*.

[3] Regan JP, Inabnet WB, Gagner M, Pomp A (2003). Early experience with two-stage laparoscopic Roux-en-Y gastric bypass as an alternative in the super-super obese patient. *Obesity Surgery*.

[4] Akkary E, Duffy A, Bell R (2008). Deciphering the sleeve: technique, indications, efficacy, and safety of sleeve gastrectomy. *Obesity Surgery*.

[5] Deitel M, Crosby RD, Gagner M (2008). The first international consensus summit for sleeve gastrectomy (SG), New York City, October 25–27, 2007. *Obesity Surgery*.

[6] Karamanakos SN, Vagenas K, Kalfarentzos F, Alexandrides TK (2008). Weight loss, appetite suppression, and changes in fasting and postprandial ghrelin and peptide-YY levels after Roux-en-Y gastric bypass and sleeve gastrectomy: a prospective, double blind study. *Annals of Surgery*.

[7] Serra C, Perez N, Bou R (2006). Gastrectomia tubular laparoscopica. Una operacion bariatrica con diferentes indicaciones. *Cirugía Española*.

[8] Marceau P, Cabanac M, Frankham PC (2005). Accelerated satiation after duodenal switch. *Surgery for Obesity and Related Diseases*.

[9] Csendes A, Braghetto I, León P, Burgos AM (2010). Management of leaks after laparoscopic sleeve gastrectomy in patients with obesity. *Journal of Gastrointestinal Surgery*.

[10] Lee CM, Cirangle PT, Jossart GH (2007). Vertical gastrectomy for morbid obesity in 216 patients: report of two-year results. *Surgical Endoscopy*.

[11] Nocca D, Krawczykowsky D, Bomans B (2008). A prospective multicenter study of 163 sleeve gastrectomies: results at 1 and 2 years. *Obesity Surgery*.

[12] Fuks D, Verhaeghe P, Brehant O (2009). Results of laparoscopic sleeve gastrectomy: a prospective study in 135 patients with morbid obesity. *Surgery*.

[13] Himpens J, Dapri G, Cadière GB (2006). A prospective randomized study between laparoscopic gastric banding and laparoscopic isolated sleeve gastrectomy: results after 1 and 3 years. *Obesity Surgery*.

[14] Braghetto I, Korn O, Valladares H (2007). Laparoscopic sleeve gastrectomy: surgical technique, indications and clinical results. *Obesity Surgery*.

[15] Csendes A, Braghetto I (2008). Sleeve gastrectomy. *Surgery Today*.

[16] Burgos AM, Braghetto I, Csendes A (2009). Gastric leak after laparoscopic-sleeve gastrectomy for obesity. *Obesity Surgery*.

[17] Cottam D, Qureshi FG, Mattar SG (2006). Laparoscopic sleeve gastrectomy as an initial weight-loss procedure for high-risk patients with morbid obesity. *Surgical Endoscopy*.

[18] Weiner RA, Weiner S, Pomhoff I, Jacobi C, Makarewicz W, Weigand G (2007). Laparoscopic sleeve gastrectomy—influence of sleeve size and resected gastric volume. *Obesity Surgery*.

[19] Han SM, Kim WW, Oh JH (2005). Results of laparoscopic sleeve gastrectomy (LSG) at 1 year in morbidly obese Korean patients. *Obesity Surgery*.

[20] Hamoui N, Anthone GJ, Kaufman HS, Crookes PF (2006). Sleeve gastrectomy in the high-risk patient. *Obesity Surgery*.

[21] Roa PE, Kaidar-Person O, Pinto D, Cho M, Szomstein S, Rosenthal RJ (2006). Laparoscopic sleeve gastrectomy as treatment for morbid obesity: technique and short-term outcome. *Obesity Surgery*.

[22] Melissas J, Koukouraki S, Askoxylakis J (2007). Sleeve gastrectomy: a restrictive procedure?. *Obesity Surgery*.

[23] Felberbauer FX, Langer F, Shakeri-Manesch S (2008). Laparoscopic sleeve gastrectomy as an isolated bariatric procedure: Intermediate-term results from a large series in three Austrian centers. *Obesity Surgery*.

[24] Tucker ON, Szomstein S, Rosenthal RJ (2008). Indications for sleeve gastrectomy as a primary procedure for weight loss in the morbidly obese. *Journal of Gastrointestinal Surgery*.

[25] Casella G, Soricelli E, Rizzello M (2009). Nonsurgical treatment of staple line leaks after laparoscopic sleeve gastrectomy. *Obesity Surgery*.

[26] Carucci LR, Turner MA, Conklin RC, DeMaria EJ, Kellum JM, Sugerman HJ (2006). Roux-en-Y gastric bypass surgery for morbid obesity: evaluation of postoperative extraluminal leaks with upper gastrointestinal series. *Radiology*.

[27] Marquez MF, Ayza MF, Lozano RB (2010). Gastric leak after laparoscopic sleeve gastrectomy. *Obesity Surgery*.

[28] Baker RS, Foote J, Kemmeter P, Brady R, Vroegop T, Serveld M (2004). The science of stapling and leaks. *Obesity Surgery*.

[29] Hamilton EC, Sims TL, Hamilton TT, Mullican MA, Jones DB, Provost DA (2003). Clinical predictors of leak after laparoscopic Roux-en-Y gastric bypass for morbid obesity. *Surgical Endoscopy*.

[30] Tan JT, Kariyawasam S, Wijeratne T, Chandraratna HS (2010). Diagnosis and management of gastric leaks after laparoscopic sleeve gastrectomy for morbid obesity. *Obesity Surgery*.

[31] Nguyen NT, Nguyen XMT, Dholakia C (2010). The use of endoscopic stent in management of leaks after sleeve gastrectomy. *Obesity Surgery*.

[32] Bege T, Emungania O, Vitton V (2011). An endoscopic strategy for management of anastomotic complications from bariatric surgery: a prospective study. *Gastrointestinal Endoscopy*.

[33] Armstrong J, O’Malley SP (2010). Outcomes of sleeve gastrectomy for morbid obesity: a safe and effective procedure?. *International Journal of Surgery*.

